# Simultaneous Maximum Likelihood Estimation for Piecewise Linear Instrumental Variable Models

**DOI:** 10.3390/e24091235

**Published:** 2022-09-02

**Authors:** Shuo Shuo Liu, Yeying Zhu

**Affiliations:** 1 Department of Statistics, The Pennsylvania State University, University Park, PA 16801, USA; 2 Department of Statistics and Actuarial Science, University of Waterloo, Waterloo, ON N2L 3G1, Canada

**Keywords:** causal inference, instrumental variables, piecewise linear, thresholds model

## Abstract

Analysis of instrumental variables is an effective approach to dealing with endogenous variables and unmeasured confounding issue in causal inference. We propose using the piecewise linear model to fit the relationship between the continuous instrumental variable and the continuous explanatory variable, as well as the relationship between the continuous explanatory variable and the outcome variable, which generalizes the traditional linear instrumental variable models. The two-stage least square and limited information maximum likelihood methods are used for the simultaneous estimation of the regression coefficients and the threshold parameters. Furthermore, we study the limiting distribution of the estimators in the correctly specified and misspecified models and provide a robust estimation of the variance-covariance matrix. We illustrate the finite sample properties of the estimation in terms of the Monte Carlo biases, standard errors, and coverage probabilities via the simulated data. Our proposed model is applied to an education-salary data, which investigates the causal effect of children’s years of schooling on estimated hourly wage with father’s years of schooling as the instrumental variable.

## 1. Introduction

In observational studies, the measured confounders can be controlled by a variety of methods such as propensity score based matching and regression adjustment. However, when the confounding variable is unmeasured, the traditional causal inference methods usually lead to biased estimators since changes in the unmeasured confounder will lead to changes in the explanatory variable, both of which will result in changes in the response variable. Failing to adjust such a confounder will lead to spurious association between the explanatory variable and the outcome. Analysis of instrumental variables (IV) has gained popularity in causal inference, such as investigating causal graphical structures [1,2] and controlling for unmeasured confounding [3,4]. An instrument is a variable that is correlated with the explanatory variable but not associated with any unmeasured confounders. In addition, the instrumental variable is supposed to have influence on the response variable only through the explanatory variable, i.e., there is no direct effect of this variable on the response. Instrumental variable analysis can be applied to many areas and disciplines, such as economics and epidemiology. For example, causality between the years of schooling and earnings in economics has been studied in the literature [5]. This example exploits the college proximity as the instrumental variable because it is revealed that those living near college or university usually have significantly higher level of education than others. On the other hand, it is believed that college proximity may improve earnings only by increasing the subject’s years of schooling. Both indicate that college proximity is a useful instrumental variable. In biomedical and epidemiological research, the main interest is to investigate the causal effect of an exposure variable on a certain disease outcome. A gene can be assumed as a good instrument if it is closely linked to the exposure but has no direct effect on the disease [6]. The study of genetic variants as instrumental variables is known as Mendelian randomization, which is discussed extensively in the literature (e.g., [7,8]). For instance, a set of 32 recently identified genetic variants are used as instrumental variables to study whether child fat mass causally affects academic achievement and blood pressure [9].

### 1.1. Related Work

Since the development of instrumental variables, a plenty of instrumental variable estimation methods have been proposed for the causal effect estimation. Two-stage least squares (2SLS) [10] is one of the most commonly used methods for the instrumental variable estimation. Theoretical analyses such as consistency and asymptotic normality also exist in the literature. When the response variable is binary, the second stage can be modified with logistic regression in mendelian randomization studies [11]. Another method is the likelihood-based method, particularly the limited information maximum likelihood (LIML) [12]. It is proved that the LIML method is more effective in dealing with the weak instruments [13]. The phenomenon of weak instruments occurs when the correlation between the instrument(s) and the explanatory variable is close to zero. When there are weak instruments, 2SLS is generally unstable and the causal effect estimators are badly biased. The typical rule of thumb to detect weak instruments is the F-statistic, which states that an instrument may be weak if the first-stage F-statistic is less than 10 [14].

Most of the IV approaches impose linear assumptions among the instrument, explanatory and response variables. However, this is not always the case. For example, a subject’s years of schooling may only have a positive effect on subsequent earnings if the subject obtained at least a high-school degree. There would be no difference in the earnings if the subject obtained either an elementary or middle school degree. In this hypothetical scenario, a linear regression model between the explanatory and response variables is clearly misspecified. When the null hypothesis of linearity relationship is rejected, one strategy could be to develop piecewise linear models, which is more interpretable compared to the completely nonlinear models.

In this paper, we propose a piecewise linear instrumental variable (PLIV) model for estimating the causal effect via a continuous threshold function. The continuous threshold function assumes that both the explanatory variable and the instrumental variable are continuous. Instrumental variable models with continuous variables have been studied extensively in the literature. For example, continuous instruments have been used in the classical IV models, developed in a structural equation modeling framework [15]. A recent paper proposes semiparametric doubly robust estimators of causal effects with the continuous instruments [16]. Moreover, some discussions about continuous exposure and a continuous response for Mendelian randomization can be found in a review paper [8].

A threshold in a variable occurs when there is a sudden change in the values of this variable. We call the point where the change happens as a cut-off point or a threshold. The subset causal effect exists when there is a threshold in the explanatory variable. The proposed PLIV model is useful because it can study the subset causal effect when the true model is not linear and it can also degenerate to a linear instrumental variable model when the relationship among the variables is indeed linear. In other words, by using piecewise linear functions, we can quantitatively find the subset effects of the explanatory and the instrumental variables.

We use the Rectified Linear Unit (ReLU) function, mathematically defined in Equation (1), to incorporate the piecewise relationships. Utilization of ReLU function for defining the subset effects have been studied in the literature, such as a regression kink model that tests the presence of the threshold [17] and the segmented and hinge models to study the subset effects in logistic regression [18]. Besides, the continuous threshold models via the ReLU function with two-way interactions is considered in the Cox’s proportional hazards model, where the asymptotic normality under mild conditions is established [19]. In this paper, we use a continuous threshold function with multiple thresholds to formulate the piecewise linear instrumental variable models. A similar study of the piecewise linear instrumental variable model through the random slope approach is studied in the literature [20]. It divides the data into a few segments and analyzes the data in each segment individually. However, this method suffers from huge efficiency and accuracy loss.

### 1.2. Contribution of This Article

In this paper, we consider a piecewise linear model when the linearity assumption of the data is inappropriate and provide a rigorous treatment of the statistical properties of the model. Our contributions can be summarized as follows.

- We simultaneously estimate the coefficients and thresholds of the piecewise linear instrumental variable model by the limited information maximum likelihood (LIML) method, assuming the number of thresholds is known.
- The proposed piecewise linear instrumental variable model will degenerate to the linear instrumental variable model if there are no thresholds. Therefore, it provides a generalization to the linear instrumental variable model. To our best knowledge, this is the first work on the piecewise linear extension to the traditional linear instrumental variable models.
- We also study the theoretical properties of the PLIV model, including the consistency and asymptotic normality of the estimators.

## 2. Piecewise Linear Instrumental Variable Model

Notations: we denote scalars by unbolded lowercase letters (e.g., sample size *n* and the *i*-th observation of outcome variable $y_{i}$), random variable by unbolded capital letter (e.g., *X*), random vectors by boldface lowercase letters (e.g., $x_{i}$ and β), and matrices with boldface capital letters (e.g., X ).

In the ordinary linear regression model $y_{i}=x_{i}^{⊤}\beta +\epsilon_{i}$, there is an assumption that the explanatory variables are uncorrelated with the error term, i.e., cov($x_{i}$,$\epsilon_{i}$) = 0. However, there are some situations where the covariance between the explanatory variables and error term exists. This leads to inconsistent estimation of ordinary least squares due to the phenomenon of endogeneity in x. One way to deal with this issue is to introduce an instrument variable, whose changes are related to changes in the explanatory variable but do not lead to the change in the response variable directly.

Let $(x_{i},y_{i},z_{i}),i=1,\cdots ,n$, denotes the observed data for (X,Y,Z), where *X* is the explanatory variable, *Y* is the response variable, and *Z* is the instrumental variable. To estimate the subset causal effect and establish the piecewise linear relationship, for any threshold parameter t∈R, we use a continuous threshold function which is defined as:

(1) $$\varphi \left(x_{i},t\right)=\left(x_{i}-t\right)I\left(x_{i}>t\right)={\left(x_{i}-t\right)}^{+},$$

where I(·) is an indicator function. ReLU function, commonly used as an activation function in deep learning, is a special case with t=0 such that $\varphi \left(x_{i},0\right)=\left(x_{i}-0\right)I\left(x_{i}>0\right)={\left(x_{i}-0\right)}^{+}$.

**Figure 1:**
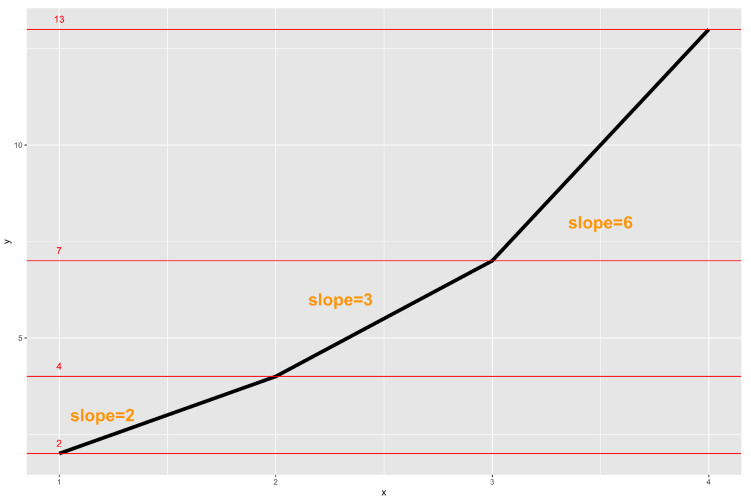
Plot of the function y=φ(x,2)+3×φ(x,3)+2x.

The proposed model provides sparsity and computational efficiency compared to the smoothing or approximation approach in the literature. The estimation stage involves indicator functions but it does not require an approximation of the indicator function. Let *K* and *J* denote the number of thresholds in *Z* and *X*, respectively. Denote $c={\left(c_{1},\cdots ,c_{K}\right)}^{T}$ as the vector of thresholds in *Z* and denote $t={\left(t_{1},\cdots ,t_{J}\right)}^{T}$ as the vector of thresholds in *X*. We propose the following piecewise linear instrumental variable model:

(2) $$x_{i}=\alpha_{0}+\alpha_{1}\varphi \left(z_{i},c_{1}\right)+\cdots +\alpha_{K}\varphi \left(z_{i},c_{K}\right)+\alpha_{K+1}z_{i}+v_{i}$$

(3) $$y_{i}=\beta_{0}+\beta_{1}\varphi \left(x_{i},t_{1}\right)+\cdots +\beta_{J}\varphi \left(x_{i},t_{J}\right)+\beta_{J+1}x_{i}+u_{i},$$

where $\beta ={\left(\beta_{0},\cdots ,\beta_{J+1}\right)}^{T}$ is the vector of coefficients representing the causal effect of *X* on *Y*; $\alpha ={\left(\alpha_{0},\cdots ,\alpha_{K+1}\right)}^{T}$ is the vector of coefficients representing the instrumental effect of *Z* on *X*; $u_{i}$ and $v_{i}$ are the error terms for the *i*th observation. In the context of causal inference, we interpret β as the causal effect of *x* on *y*. More specifically, for $t_{j}<x\leq t_{j+1},1\leq j\leq J$ with $t_{J+1}$ denoting the maximum value of *x*, one unit increase in *x* leads to $\beta_{J+1}+\sum_{j^{\prime }=1}^{j}\beta_{j^{\prime }}$ units change in *y*. Besides, $\beta_{J+1}$ represents the change in *y* that is caused by one unit increase in *x* for $t_{0}<x\leq t_{1}$ where $t_{0}$ is the minimum value of *x*. To better understand this, in Figure 1, we plot the function y=φ(x,2)+3×φ(x,3)+2x where $\beta_{1}=1,\beta_{2}=3,\beta_{3}=2$ as an example. When 2<x≤3, the slope is $\beta_{1}+\beta_{3}=3$. When 3<x≤4, the slope is $\beta_{1}+\beta_{2}+\beta_{3}=6$.

Here, we assume *K* and *J* are prespecified according to some prior knowledge or theoretical justifications. Practically, we may use the Akaike information criterion (AIC) or the Bayesian information criterion (BIC) [21] to select them. A more elegant examination of the condition for the number of thresholds can be found in Newey [22]. In particular, when $\alpha_{1}=\cdots =\alpha_{K}=0$ and $\beta_{1}=\cdots =\beta_{J}=0$, our proposed model degenerates to the traditional linear instrumental variable model.

For instrumental variable analysis, an instrumental variable is correlated with the explanatory variable but not correlated with the error term. In our model, ${\left(Z-c\right)}^{+}=\left\{{\left(Z-c_{1}\right)}^{+},\cdots ,{\left(Z-c_{K}\right)}^{+}\right\}$ is the vector of instrumental variables with the following properties:

- Instrument relevance: $\mathrm{cov}\{{\left(Z-c\right)}^{+},X\}\neq 0$: ${\left(Z-c\right)}^{+}$ is correlated with the explanatory variable *X*.
- Instrument exogeneity: $\mathrm{cov}\{{\left(Z-c\right)}^{+},U\}=0$: ${\left(Z-c\right)}^{+}$ is uncorrelated with the error term *U*.

We assume K≥J for identifiability, i.e., the number of instruments should be larger than or equal to the number of endogenous variables.

**Remark** **1.** *Note that intensive research about nonlinear instrumental variable models has been conducted in the literature, such as the nonparametric instrumental regression [23,24,25]. We point out that the target of our method is to quantitatively find the thresholds and estimate the subset causal effects. We aim to generalize the traditional linear IV model and fit an interpretable model rather than approximate the data by a nonlinear function.*

To estimate the unknown parameters in (2) and (3), we utilize the two-stage least square (2SLS) method and the limited information maximum likelihood (LIML) method. Details about the proposed estimation methods are discussed below.

## 3. Simultaneous Maximum Likelihood Estimation

We first introduce how the LIML method is used in our model and initialize the naive estimators by the 2SLS method.

### 3.1. Limited Information Maximum Likelihood

As discussed in the introduction about the advantages, limited information maximum likelihood is another popular approach for estimation in the instrumental variable models. Here, we assume the error terms (U,V) are jointly normally distributed and correlated to some extent due to the unmeasured confounding effect. Let 0 be the zero-mean vector and ρ be the correlation of (U,V). Denote $\sigma_{u}^{2}$ and $\sigma_{v}^{2}$ as the variance of the error terms *U* and *V*, respectively. Then the probability density function of the bivariate normal (U,V) can be written as:

$$f\left(U,V\right)=\frac{1}{2\pi \sigma_{u}\sigma_{v}\sqrt{1-\rho^{2}}}\exp\left[-\frac{1}{2(1-\rho^{2})}Q\left(U,V\right)\right],$$

where the quadratic form $Q\left(U,V\right)=\frac{U^{T}U}{\sigma_{u}^{2}}-\frac{2\rho U^{T}V}{\sigma_{v}\sigma_{u}}+\frac{V^{T}V}{\sigma_{v}^{2}}$. For a single observation, the log-likelihood is

$$\ell \left(u_{i},v_{i};\theta \right)\propto -\log\left(\sigma_{u}\sigma_{v}\right)-\frac{1}{2}\log\left(1-\rho^{2}\right)-\frac{1}{2(1-\rho^{2})}\left(\frac{u_{i}^{2}}{\sigma_{u}^{2}}-\frac{2\rho u_{i}v_{i}}{\sigma_{u}\sigma_{v}}+\frac{v_{i}^{2}}{\sigma_{v}^{2}}\right),$$

where $\theta ={\left(\alpha^{T},\beta^{T},c^{T},t^{T},\rho ,\sigma_{u},\sigma_{v}\right)}^{T}$ denote all the model parameters and

$$v_{i}=x_{i}-\alpha_{0}-\alpha_{1}\varphi \left(z_{i},c_{1}\right)-\cdots -\alpha_{K}\varphi \left(z_{i},c_{K}\right)-\alpha_{K+1}z_{i}$$

$$u_{i}=y_{i}-\beta_{0}-\beta_{1}\varphi \left(x_{i},t_{1}\right)-\cdots -\beta_{J}\varphi \left(x_{i},t_{J}\right)-\beta_{J+1}x_{i}.$$

To simplify notations, we let $\ell \left(\theta \right)=\ell \left(u_{i},v_{i};\theta \right)$ denote the log-likelihood. The maximum likelihood estimates for θ is obtained by maximizing the log-likelihood within the compact set $\Theta \subset R^{D(\theta )}$ such that ${\hat{\theta }}_{n}={arg max}_{\theta \in \Theta }\ell_{n}\left(\theta \right)$, where $\ell_{n}\left(\theta \right)=1/n\sum_{i=1}^{n}\ell \left(\theta \right)$. However, there is no closed-form solution for θ, so we take the gradient-based algorithm for estimation. This yields approximate M-estimators. To speed up estimation, we use the two-stage least square method to initialize the estimators.

### 3.2. Initialization: Two-Stage Least Square

The traditional two-stage least squares method regresses the explanatory variable on the instrumental variable and computes the predictions $\hat{x}$ in the first stage. In the second stage, it regresses the response variable on the predictions $\hat{x}$. The causal effect of interest is estimated from the second stage. In our method, we employ 2SLS to obtain the initial values of the parameters of the piecewise linear instrumental variable model. Below we describe the 2SLS procedures for initializations:

Stage 1: First, we regress *x* on $\left\{{\left(z-c\right)}^{+},z\right\}$ and then obtain the fitted values $\hat{x}$, where ${\left(z-c\right)}^{+}=\left\{{\left(z-c_{1}\right)}^{+},\cdots ,{\left(z-c_{K}\right)}^{+}\right\}$.

Stage 2: We regress *y* on $\left\{{\left(\hat{x}-t\right)}^{+},\hat{x}\right\}$, where ${\left(\hat{x}-t\right)}^{+}=\left\{{\left(\hat{x}-t_{1}\right)}^{+},\cdots ,{\left(\hat{x}-t_{J}\right)}^{+}\right\}$. Thus, in the second stage, we fit the following regression model:

$$y_{i}=\beta_{0}+\beta_{1}\varphi \left({\hat{x}}_{i},t_{1}\right)+\cdots +\beta_{J}\varphi \left({\hat{x}}_{i},t_{J}\right)+\beta_{J+1}{\hat{x}}_{i}+u_{i}.$$

For each combination of the number of thresholds in *X* and *Z*, we could pick c, t and the regression coefficients simultaneously through grid search when the sum of squared errors (SSE) of Y is minimized. However, for J≥2 or K≥2, it is slightly computationally expensive to conduct grid search. Since we only need 2SLS to provide the initialization of the parameters in our method, we choose c to be a vector of the points that are evenly spaced between the 5% to 95% quantiles of *Z*. Similarly, we choose t to be a vector of the points that are evenly spaced between the 5% to 95% quantiles of *X*. We ignore points below and above the 5% to 95% quantiles in order to avoid boundary effects. The regression coefficients are obtained accordingly.

### 3.3. Theoretical Analysis

Under mild conditions, we study the statistical properties of the proposed model and establish the robust variance-covariance estimators for the estimated parameters under the correctly specified and misspecified models, separately. To investigate the theoretical properties, we consider the following regularity conditions:

- C1. Observations $(X_{i},Y_{i},Z_{i}),i=1,\cdots ,n$ are independently and identically distributed on a compact set $X⊗Y⊗Z\subset R^{1}⊗R^{1}⊗R^{1}$. Furthermore, $E(∥X{∥}^{2})<\infty$, $E(∥Y{∥}^{2})<\infty$, and $E(∥Z{∥}^{2})<\infty$.
- C2. The explanatory variable *X* and the instrumental variable *Z* are continuous in the parameter space, i.e., they have continuous probability density functions $f_{X}\left(\cdot \right)$ and $f_{Z}\left(\cdot \right)$. The density functions are uniformly bounded, that is, there exist constants ${\underline{c}}_{1}$, ${\underline{c}}_{2}$, ${\bar{c}}_{1}$, and ${\bar{c}}_{2}$ such that
  $${\underline{c}}_{1}\leq \underset{Z\in Z}{\inf}f_{Z}\left(\cdot \right)\leq \underset{Z\in Z}{\sup}f_{Z}\left(\cdot \right)\leq {\bar{c}}_{1}\;\mathrm{and}\;{\underline{c}}_{2}\leq \underset{X\in X}{\inf}f_{X}\left(\cdot \right)\leq \underset{X\in X}{\sup}f_{X}\left(\cdot \right)\leq {\bar{c}}_{2}.$$
  Furthermore, the true value of the coefficients for the threshold effects satisfy $\alpha_{0}^{-}\neq 0$ and $\beta_{0}^{-}\neq 0$, where $\alpha_{0}^{-}=\left(\alpha_{20},\cdots ,\alpha_{(K-1)0}\right)$ and $\beta_{0}^{-}=\left(\beta_{20},\cdots ,\beta_{(J-1)0}\right)$.
- C3. ℓ(θ) is upper-semicontinuous for almost all (X,Y,Z), that is, for every θ,
  $$\underset{\theta_{n}\to \theta }{lim sup}\ell \left(X,Y,Z;\theta_{n}\right)\leq \ell \left(X,Y,Z;\theta \right),\;a.s.$$

**Remark** **2.** *Condition C1 is commonly used in regression models. Condition C2 is used for estimating the unknown thresholds and ensures the model is identifiable. The continuity requirements of X and Z are used to estimate the thresholds. Condition C3 is used to establish the consistency and the asymptotic normality of the maximum likelihood estimator.*

In terms of estimation, we take the gradient-based method which depends on the first order derivative $\dot{\ell }\left(\theta \right)=\partial \ell \left(\theta \right)/\partial \theta$ (details can be found in Appendix A) with the initialized estimators by 2SLS. In this paper, we do not approximate the indicator function by the logistic function as some researchers do (e.g., [18,26,27]). The gradient-based algorithm for the ReLU function has shown success in the context of deep learning and machine learning. Compared to the approximation techniques as discussed in Section 1, model estimation with the ReLU function is computationally cheaper since no approximation of the indicator function is required. In fact, as long as Condition C2 is satisfied which requires variables X and Z to be continuous, the gradients composed of the indicator functions converge to a continuous function of the threshold parameters as n→∞, for example,

$$\frac{1}{n}\sum_{i=1}^{n}I\left(z_{i}>c_{k}\right)\overset{P}{\to }E\left\{I(z_{i}>c_{k})\right\}=P\left(z_{i}>c_{k}\right),$$

for k=1,⋯,K by the law of large numbers. Therefore, the second order derivative of the ReLU function with respect to the thresholds can be derived based on the resulting continuous probability function. More specifically, the second order derivative with respect to $c_{k}$ is simply $-f_{Z}\left(c_{k}\right)$.

To prove the asymptotic normality, we first need to show the consistency of the proposed estimators.

**Theorem** **1.** *Under conditions C1–C4, assume that Θ is compact and the true parameter vector $\theta_{0}={arg max}_{\theta \in \Theta }E\left\{\ell (\theta )\right\}$ is unique. Furthermore, for every sufficiently small ball B⊂Θ, $\sup_{\theta \in B}\ell \left(\theta \right)$ is measurable with $E\sup_{\theta \in B}\ell \left(\theta \right)<\infty$, then ${\hat{\theta }}_{n}\overset{p}{\to }\theta_{0}.$*

**Proof.**  The proof follows the Theorem 5.7 of van der Vaart [28]. For completeness, we include it as Theorem A1 in Appendix B. To utilize Theorem 5.7, we need to check the condition that $\ell \left({\hat{\theta }}_{n}\right)\geq \ell \left(\theta_{0}\right)-o_{P}\left(1\right)$ for some $\theta_{0}\in \Theta_{0}$. This is true since $\ell_{n}\left(\theta \right)$ is continuous in θ, $\ell_{n}\left(\theta \right)$ converges to ℓ(θ) uniformly, and ${\hat{\theta }}_{n}$ (approximately) maximizes $\ell_{n}\left(\theta \right)$. Thus, all the conditions are satisfied and the result follows. □

**Theorem** **2.** *Under conditions C1–C4, let $\theta_{0}$ be the true value of θ. Let $\dot{\ell }\left(\theta \right)$ be a measurable function with $E\left[{\left\{\dot{\ell }\left(\theta \right)\dot{\ell }{\left(\theta \right)}^{T}\right\}}_{\left(i,j\right)}\right]<\infty$ for $i,j={1,\cdots ,|\theta |}_{*}$, where ${\left|\theta \right|}_{*}$ denotes the number of elements in θ, then*

$$\sqrt{n}\left({\hat{\theta }}_{n}-\theta_{0}\right)\overset{d}{\to }N\left(0,V_{\theta_{0}}^{-1}M_{\theta_{0}}V_{\theta_{0}}^{-1}\right),$$

*where $M_{\theta_{0}}=E\left\{\dot{\ell }\left(\theta_{0}\right)\dot{\ell }{\left(\theta_{0}\right)}^{T}\right\}$ and $\dot{\ell }\left(\theta_{0}\right)$ is the first order derivative of ℓ(θ) with respect to θ evaluated at $\theta_{0}$ and $V_{\theta_{0}}$ is the second order derivative of E{ℓ(θ)} with respect to θ evaluated at $\theta_{0}$ (derivations in Appendix A). $V_{\theta }$ has the form*

$$V_{\theta }=V_{\theta }^{\left(1\right)}+V_{\theta }^{\left(2\right)}=V_{\theta }^{\left(1\right)}+\left(\begin{array}{ccccccc} 0 & 0 & V_{\alpha c}^{\left(2\right)} & 0 & 0 & 0 & 0\\ & 0 & 0 & V_{\beta t}^{\left(2\right)} & 0 & 0 & 0\\ & & V_{cc}^{\left(2\right)} & 0 & 0 & 0 & 0\\ & & & V_{tt}^{\left(2\right)} & 0 & 0 & 0\\ & & & & 0 & 0 & 0\\ & & & & & 0 & 0\\ \mathrm{sym}. & & & & & & 0 \end{array}\right),$$

*where 0 denotes a zero vector or a zero matrix and 0 denotes a scalar. Details of $V_{\theta }^{\left(1\right)}$ and $V_{\theta }^{\left(2\right)}$ are given in the Appendix A.*

**Proof.**  First, note that ℓ(θ) is Lipschitz continuous in θ. Moreover, the fact that $V_{\theta }$ is continuous in θ admits the Taylor expansion of $E_{XYZ}\ell \left(\theta \right)$:

$$E_{\left(X,Y,Z\right)}\ell \left(\theta \right)=E_{\left(X,Y,Z\right)}\ell \left(\theta_{0}\right)+\frac{1}{2}\left(\theta -\theta_{0}\right)V_{\theta_{0}}{\left(\theta -\theta_{0}\right)}^{T}+o_{p}\left({∥\theta -\theta_{0}∥}^{2}\right).$$

Since $\hat{\theta }$ is the maximum likelihood estimate of θ, $\frac{1}{n}\sum_{i=1}^{n}\ell \left(\hat{\theta }\right)\geq \sup_{\theta }\frac{1}{n}\sum_{i=1}^{n}\ell \left(\theta \right)-o_{P}\left(\frac{1}{n}\right)$. Plus the result from Theorem 1 that ${\hat{\theta }}_{n}\overset{p}{\to }\theta_{0}$, we conclude from Theorem 5.14 of van der Vaart [28] that:

$$\sqrt{n}\left({\hat{\theta }}_{n}-\theta_{0}\right)=-V_{\theta_{0}}^{-1}\frac{1}{\sqrt{n}}\sum_{i=1}^{n}{\dot{\ell }}_{i}\left(\theta_{0}\right)+o_{P}\left(1\right),$$

which implies an asymptotic normal distribution with mean 0 and variance-covariance matrix $V_{\theta_{0}}^{-1}M_{\theta_{0}}V_{\theta_{0}}^{-1}$. □

For completeness, we include Theorem 5.14 of van der Vaart [28] (2000) as Theorem A2 in Appendix B. When the model is correctly specified, $V_{\theta_{0}}=-M_{\theta_{0}}$, the asymptotic variance is the inverse of Fisher information. Matrices $V_{\theta_{0}}$ and $M_{\theta_{0}}$ are estimated through the replacement of $\theta_{0}$ by the MLE ${\hat{\theta }}_{n}$. Thus, for the correctly specified model, the variance-covariance matrix is estimated by the inverse of $M_{{\hat{\theta }}_{n}}$. For the misspecified model, the variance-covariance matrix is estimated by $V_{{\hat{\theta }}_{n}}^{-1}M_{{\hat{\theta }}_{n}}V_{{\hat{\theta }}_{n}}^{-1}$. Let us define $V_{n}$ as the second derivative of $\ell_{n}\left(\theta \right)$ with respect to θ, then we can decompose $V_{n}$ the same way as $V_{\theta }$ into two matrices $V_{n}^{\left(1\right)}$ and $V_{n}^{\left(2\right)}$. Note that $V_{n}$ is the empirical process of $V_{\theta }$ and $V_{n}\overset{p}{\to }V_{\theta }$ by the law of large numbers, so we use the estimated probability densities ${\hat{f}}_{Z}\left({\hat{c}}_{k}\right)$ and ${\hat{f}}_{X}\left({\hat{t}}_{j}\right)$ for $f_{Z}\left(c_{k}\right)$ and $f_{X}\left(t_{j}\right)$ for k=1,⋯,K and j=1,⋯,J, respectively.

## 4. Simulation Studies

In this section, we evaluate the performance of the proposed model using simulated datasets. We consider two scenarios with the same sample size n=500. We let error terms *U* and *V* be jointly normally distributed with mean 0 and correlation ρ∈{0.2,0.5,0.8}. Here, we consider a common standard deviation $\sigma_{u}=\sigma_{v}=\sqrt{0.3}$. Besides, we simulate the instrumental variable Z∼N(0,1). The first scenario has one threshold in *X* and one threshold in *z*, and it takes the following form:

$$\begin{array}{cc} x_{i} & =-1+0.5\times {\left(z_{i}-0.5\right)}^{+}+z_{i}+v_{i}\\ y_{i} & =-0.2+{\left(x_{i}-0\right)}^{+}+0.5\times x_{i}+u_{i}. \end{array}$$

The true values of the parameters in PLIV models are α=(−1,0.5,1), β=(−0.2,1,0.5), c=0.5, and t=0. The second scenario has two thresholds in *x* and two thresholds in *z*, and it takes the following form:

$$\begin{array}{cc} x_{i} & =-1+0.5\times {\left(z_{i}+1\right)}^{+}+{\left(z_{i}-1\right)}^{+}+z_{i}+v_{i}\\ y_{i} & =-1+1.2\times {\left(x_{i}+1\right)}^{+}+{\left(x_{i}-2\right)}^{+}+0.5\times x_{i}+u_{i}. \end{array}$$

**Figure 2:**
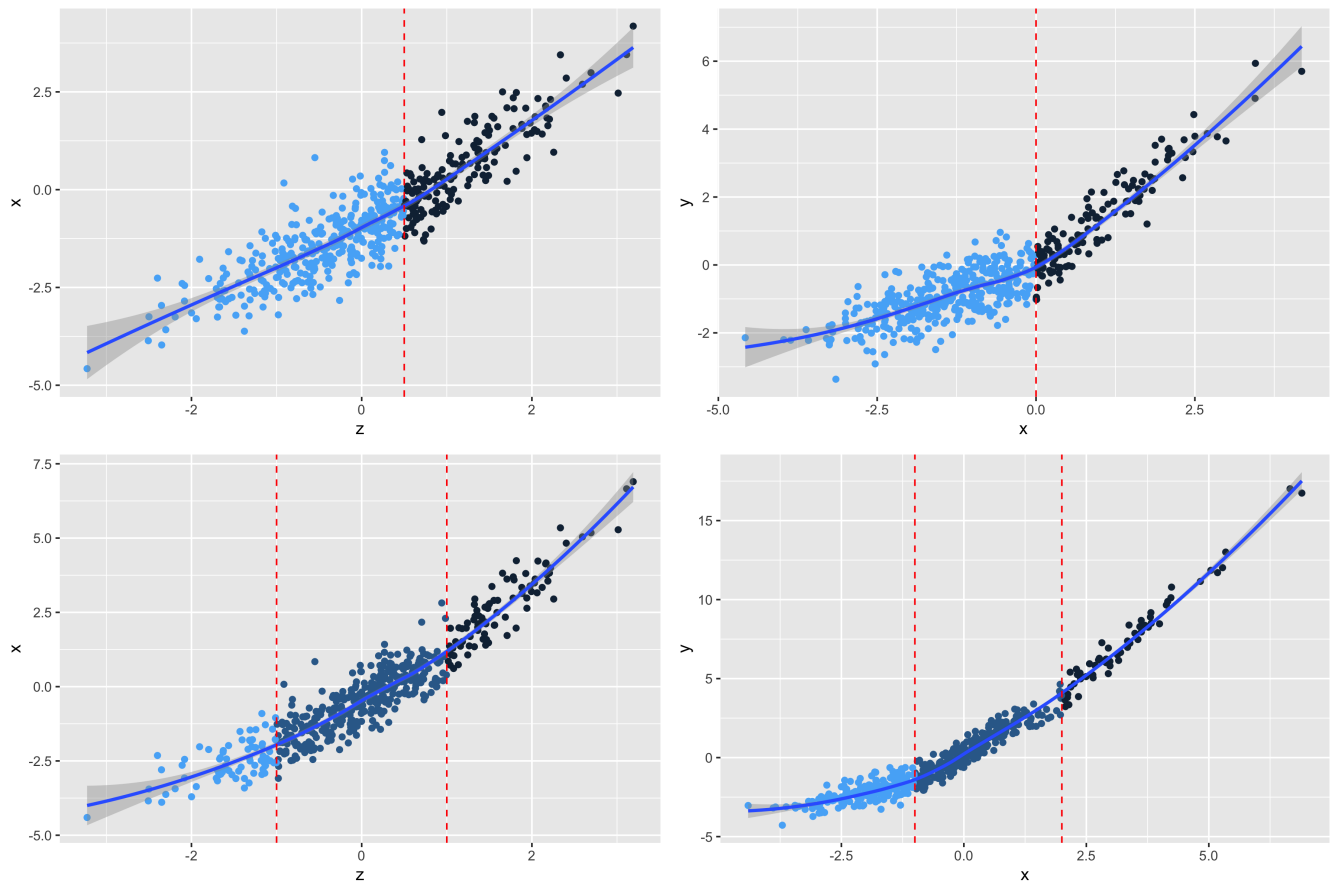
Piecewise linear instrumental variable models with simulated data for scenario 1 and scenario 2. The upper panel plots the simulated *X* versus *Z*, *Y* versus *X* for scenario 1, respectively. The lower panel plots the simulated *X* versus *Z*, *Y* versus *X* for scenario 2, respectively.

The true parameters are α=(−1,0.5,1,1), β=(−1,1.2,1,0.5), c=(−1,1), and t=(−1,2). We show the simulated piecewise linear instrumental variable models for scenario 1 and scenario 2 in Figure 2. We replicate the simulation 1000 times to evaluate the finite sample properties of the proposed model by the PLIV method.

Table 1 summarizes the biases, standard errors of $\hat{\theta }$ and coverage probabilities of θ by the proposed PLIV method for scenario 1, where tse is the theoretical standard error and ese is the empirical standard error. As we can see in the table, all the biases of $\hat{\theta }$ are close to zero. We also find that the theoretical standard error and the empirical standard error are close enough, which confirms the validity of our theoretical results in Section 3. The results show that our model estimation is quite accurate and therefore provides unbiased and consistent estimators. Besides, we notice that the coverage probabilities are around 95% under different values of ρ. Moreover, biases and the standard errors decrease as we increase ρ because the instrumental variables becomes stronger.

Table 2 summarizes the biases, standard errors of $\hat{\theta }$ and 95% coverage probabilities of θ by the PLIV method for scenario 2, where tse is the theoretical standard error and ese is the empirical standard error. We find the similar patterns as in Table 1 from scenario 1. For instance, all the biases are small. Theoretical standard errors and the empirical standard errors are close to each other. Most coverage probabilities are around 95% when ρ=0.2 and ρ=0.5. We also observe that the coverage probabilities of the thresholds are slightly low when ρ=0.8. The reason might be due to the high correlation between errors. With multiple thresholds and high correlation, it poses challenges to estimate the exact locations.

We include results with a sample size of 1000 in Appendix C, while fixing ρ=0.5. Overall, as *n* increases, we observe that both biases and standard errors drop.

## 5. Application

In this section, we revisit the Card’s education data [5]. We apply the proposed model to study the causal effect of years of schooling on hourly wage in cents with father’s years of schooling as the instrumental variable. The interest here is to find a threshold and study the threshold effect of the years of schooling. It is generally believed that a child’s years of schooling has a direct effect on the child’s wage and parents’ education only affects the child’s income by affecting the child’s education level. In other words, parents’ education level has no direct effect on child’s wage. Therefore, the father’s years of schooling can be treated as a valid instrumental variable.

**Figure 3:**
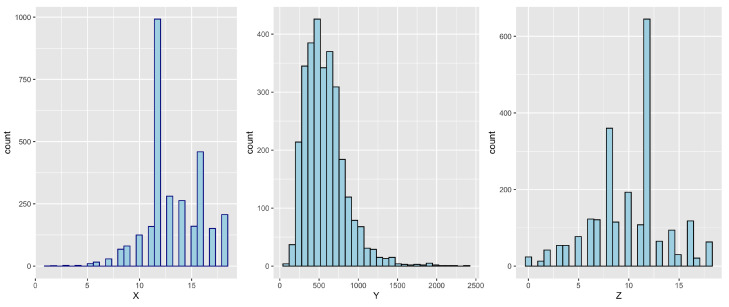
Histogram plots of the raw data *X*, *Y*, and *Z*.

In Card’s data, we remove the missing values and include a total of n=2657 observations. The explanatory variable *X* (child’s years of education) is between 1 and 18 with median 13, and the instrumental variable *Z* (father’s years of education) has minimum 0, maximum 18, and median 12. Figure 3 indicates that variables *X* and *Y* are skewed and have heavy tails so transformations are needed before the analysis. A log transformation is applied to both.

Table 3 shows the point estimate, standard error, and associated 95% confidence interval of θ by the proposed model with K=1 and J=0, which are selected by BIC. In the table, $\alpha_{1}$ and *c* are the coefficient and threshold for the transformed father’s years of schooling, respectively. $\beta_{1}$ is the causal effect of years of schooling on earnings. The estimated causal effect of interest ${\hat{\beta }}_{1}$ is 0.87, which results in a difference of exp(0.87×a) units increase in wage if there are *a* units increase in the log of years of schooling. In economics, ${\hat{\beta }}_{1}$ is interpreted as “elasticity". That is, if years of education increases by 1%, the person’s income will increase by 0.87% by our estimation. In terms of the instrumental variable, we notice that the threshold *c* is estimated to be 7.86. The corresponding p-value is not calculated since testing c=0 is meaningless in this context. It shows that there exists a threshold at around 8 in the father’s years of schooling. That is, the father’s years of schooling only has a positive effect on the child’s years of schooling if father receives at least 8 years of education. This information can not be observed if the traditional 2SLS method or nonparametric approaches are applied to analyze the data. The threshold effect as well as the thresholds are all statistically significant since their corresponding p-values are far less than 0.05.

## 6. Discussion, Limitations, and Future Research

In this paper, we propose a simultaneous maximum likelihood estimation for a piecewise linear instrumental variable model. We use the two-stage least square estimators as the initial values and the limited information maximum likelihood methods to estimate the regression coefficients and the threshold parameters simultaneously. We also provide a robust inference of the proposed model. The proposed model with the piecewise linear functions allows us to find the thresholds for both the explanatory and the instrumental variables, which generalizes the traditional linear instrumental variable models. In the simulation study, we evaluate the performance of the proposed model and find that it behaves well in terms of the biases, standard errors, and coverage probabilities in different settings.

In our model, we include a single continuous explanatory variable and a single continuous instrumental variable. We assume the explanatory variable and the instrumental variable are continuous. More complicated cases can be considered. For example, developing a piecewise linear model with count data might be interesting. However, finding the optimal number of thresholds as well as the locations is challenging from the theoretical side. Furthermore, we assume the number of thresholds *K* and *J* are prespecified. Treating the numbers of thresholds as random variables, finding the optimal values, and investigating the theoretical properties can be future research.

## Figures and Tables

**Table 1:** Empirical biases, theoretical standard errors (tse), and empirical standard errors (ese) of $\hat{\theta }$, as well as 95% coverage probabilities (cp) on θ for scenario 1.

	ρ=0.2				ρ=0.5				ρ=0.8			
	bias	tse	ese	cp	bias	tse	ese	cp	bias	tse	ese	cp
$\alpha_{0}$	−19.25	41.25	45.80	937	−16.43	38.26	41.56	939	−9.10	32.08	33.78	940
$\alpha_{1}$	7.65	98.27	102.66	927	6.36	93.13	97.02	924	4.10	77.32	81.80	919
$\alpha_{2}$	−16.95	46.20	47.71	931	−14.79	42.82	43.64	933	−8.28	33.52	34.34	943
$\beta_{0}$	−7.86	55.41	54.87	950	−6.88	52.37	52.74	944	−4.28	43.92	44.80	945
$\beta_{1}$	0.48	80.58	77.07	955	−0.35	75.48	74.69	942	−0.58	60.37	62.50	940
$\beta_{2}$	−4.35	34.57	34.06	947	−3.84	32.49	32.60	945	−2.38	26.21	26.57	933
*c*	−95.15	178.21	247.82	839	−82.89	159.34	224.83	846	−46.25	113.96	165.49	864
*t*	−14.88	97.77	108.77	922	−12.71	87.80	101.10	908	−6.76	62.69	71.68	908
ρ	2.82	48.99	47.54	951	2.67	37.91	36.81	947	1.62	17.70	17.22	941
$\sigma^{2}$	−2.32	14.00	13.72	954	−1.85	15.65	15.40	953	−1.10	18.12	17.82	956

Note: all numbers are multiplied by 1000. These results are based on 1000 replications.

**Table 2:** Empirical biases, theoretical standard errors (tse), and empirical standard errors (ese) of $\hat{\theta }$, as well as 95% coverage probabilities (cp) on θ for scenario 2.

	ρ=0.2				ρ=0.5				ρ=0.8			
	bias	tse	ese	cp	bias	tse	ese	cp	bias	tse	ese	cp
$\alpha_{0}$	−51.88	268.22	247.08	946	−38.92	232.37	226.53	939	−20.83	158.06	169.46	921
$\alpha_{1}$	29.20	176.58	157.46	966	24.67	157.87	143.26	965	13.44	110.56	107.65	949
$\alpha_{2}$	15.11	172.47	166.40	943	11.80	178.03	163.63	949	11.40	146.19	143.76	955
$\alpha_{3}$	−26.32	164.95	147.35	945	−19.39	144.98	135.53	931	−9.21	101.13	101.32	934
$\beta_{0}$	−8.36	120.42	116.63	944	−8.23	111.05	108.00	950	−0.84	85.31	82.56	958
$\beta_{1}$	6.61	71.82	71.49	947	6.57	66.84	66.57	948	3.39	52.07	52.12	950
$\beta_{2}$	6.44	115.13	99.07	966	5.38	106.29	90.78	969	3.30	83.05	75.06	962
$\beta_{3}$	−4.14	57.89	56.20	947	−4.33	53.69	52.40	950	−1.10	41.80	40.31	955
$c_{1}$	−3.01	253.38	246.83	930	9.41	221.21	257.36	924	6.90	152.06	218.68	898
$c_{2}$	2.15	120.17	138.80	913	5.07	139.96	140.17	901	9.10	84.42	134.44	880
$t_{1}$	0.79	76.25	79.60	944	1.04	68.31	72.98	939	4.57	48.70	49.52	935
$t_{2}$	18.65	168.54	189.81	926	17.60	149.74	174.54	911	16.26	104.90	158.56	922
ρ	2.87	47.44	45.58	950	3.40	36.81	35.35	953	2.14	17.37	16.77	948
$\sigma^{2}$	−3.64	14.00	13.64	939	−2.99	15.55	15.21	946	−1.84	17.99	17.63	955

Note: all numbers are multiplied by 1000. These results are based on 1000 replications.

**Table 3:** Summary table of θ by the SML-PLIV model.

Parameter	Estimate	Std. Error	z Value	95% C.I.	*p*-Value
$\alpha_{0}$: intercept	2.25	0.013	168.8	(2.222, 2.274)	≈0
$\alpha_{1}$: ${\left(Z-c\right)}^{+}$	−0.02	0.003	−4.8	(−0.023, −0.009)	≈0
$\alpha_{2}$: *Z*	0.04	0.003	14.3	(0.033, 0.043)	≈0
$\beta_{0}$: intercept	4.04	0.217	18.6	(3.613, 4.464)	≈0
$\beta_{1}$: logX	0.87	0.084	10.4	(0.705, 1.033)	≈0
*c*	7.86	0.939	8.4	(6.016, 9.696)	-

## Data Availability

Data used in the application section come from the ivmodel package of CRAN, which can be downloaded from https://github.com/hyunseungkang/ivmodel/tree/master/data (accessed on 31 August 2022). Codes to simulate data, generate tables and plots in Section 4 can be found at https://github.com/shuoshuoliu/PLIV (accessed on 31 August 2022).

## Appendix A. Derivation of the Information and Hessian Matrices

The likelihood to be minimized is

$$\ell_{\theta }=\frac{1}{n}\sum_{i=1}^{n}\left\{-\log\left(\sigma_{u}\sigma_{v}\right)-\frac{1}{2}\log\left(1-\rho^{2}\right)-\frac{1}{2(1-\rho^{2})}\left(\frac{u_{i}^{2}}{\sigma_{u}^{2}}-\frac{2\rho u_{i}v_{i}}{\sigma_{u}\sigma_{v}}+\frac{v_{i}^{2}}{\sigma_{v}^{2}}\right)\right\}.$$

When the model is specified,

$$E_{XYZ}\ell_{\theta }=-\log\left(\sigma_{u}\sigma_{v}\right)-\frac{1}{2}\log\left(1-\rho^{2}\right)-\frac{1}{2(1-\rho^{2})}E_{XYZ}\left(\frac{U^{T}U}{\sigma_{u}^{2}}-\frac{2\rho U^{T}V}{\sigma_{v}\sigma_{u}}+\frac{V^{T}V}{\sigma_{v}^{2}}\right).$$

To write out the first order derivative $\dot{\ell }\left(\theta \right)$ of $\ell_{\theta }$ with respect to θ, we define the following notations. $\partial \ell_{\theta }/\partial \alpha c$ is the row concatenation of the first order derivative of $\ell_{\theta }$ with respect to α and c. $\partial \ell_{\theta }/\partial \beta t$ is the row concatenation of the first order derivative of $\ell_{\theta }$ with respect to β and t. For notation simplicity, we drop the subscription *i*. Let $\alpha I\left(z>c\right)=\{\alpha_{1}I\left(z>c_{1}\right),\cdots ,\alpha_{k}I\left(z>c_{K}\right)\}$ and $\beta I\left(x>t\right)=\{\beta_{1}I\left(x>t_{1}\right),\cdots ,\beta_{j}I\left(x>t_{J}\right)\}$. Then we can divide the first order derivative $\dot{\ell }\left(\theta \right)$ as following

(A1) $$\begin{array}{c} \frac{\partial \ell_{\theta }}{\partial \alpha c}=\frac{1}{n}\sum_{i=1}^{n}\left[{\left\{1,{\left(z-c\right)}^{+},z,-\alpha I\left(z>c\right)\right\}}^{T}\frac{1}{\left(1-\rho^{2}\right)}\left(\frac{v}{\sigma_{v}^{2}}-\frac{\rho u}{\sigma_{u}\sigma_{v}}\right)\right]\\ \frac{\partial \ell_{\theta }}{\partial \beta t}=\frac{1}{n}\sum_{i=1}^{n}\left[{\left\{1,{\left(x-t\right)}^{+},x,-\beta I\left(x>t\right)\right\}}^{T}\frac{1}{\left(1-\rho^{2}\right)}\left(\frac{u}{\sigma_{u}^{2}}-\frac{\rho v}{\sigma_{u}\sigma_{v}}\right)\right]\\ \frac{\partial \ell_{\theta }}{\partial \rho }=\frac{1}{n}\sum_{i=1}^{n}\left[\frac{\rho }{1-\rho^{2}}-\frac{\rho }{{\left(1-\rho^{2}\right)}^{2}}\left(\frac{u^{2}}{\sigma_{u}^{2}}-\frac{2\rho uv}{\sigma_{u}\sigma_{v}}+\frac{v^{2}}{\sigma_{v}^{2}}\right)+\frac{uv}{\sigma_{v}\sigma_{u}\left(1-\rho^{2}\right)}\right]\\ \frac{\partial \ell_{\theta }}{\partial \sigma_{u}}=\frac{u^{2}}{\left(1-\rho^{2}\right)\sigma_{u}^{3}}-\frac{\rho uv}{\left(1-\rho^{2}\right)\sigma_{v}\sigma_{u}^{2}}-\frac{1}{\sigma_{u}}\\ \frac{\partial \ell_{\theta }}{\partial \sigma_{v}}=\frac{v^{2}}{\left(1-\rho^{2}\right)\sigma_{v}^{3}}-\frac{\rho uv}{\left(1-\rho^{2}\right)\sigma_{u}\sigma_{v}^{2}}-\frac{1}{\sigma_{v}} \end{array}.$$

The interchangeability of expectation and differentiation is satisfied here and it implies $\partial E_{XYZ}\ell \left(\theta \right)/\partial \theta =E_{XYZ}\left\{\dot{\ell }\left(\theta \right)\right\}$. It is easy to check $\partial E_{XYZ}\ell_{\theta }/\partial \theta =0$ at $\theta_{0}$ as it should be. We next derive the second order derivative $V_{\theta }$ of $E_{XYZ}\ell_{\theta }$ when the model is specified. We partition the symmetric matrix $V_{\theta }$ as two symmetric matrices $V_{1,\theta }$ and $V_{2,\theta }$ such that

$$\begin{array}{c} V_{\theta }=V_{\theta }^{\left(1\right)}+V_{\theta }^{\left(2\right)}=V_{\theta }^{\left(1\right)}+\left(\begin{array}{ccccccc} 0 & 0 & V_{\alpha c}^{\left(2\right)} & 0 & 0 & 0 & 0\\ \; & 0 & 0 & V_{\beta t}^{\left(2\right)} & 0 & 0 & 0\\ \; & \; & V_{cc}^{\left(2\right)} & 0 & 0 & 0 & 0\\ \; & \; & & V_{tt}^{\left(2\right)} & 0 & 0 & 0\\ \; & \; & & \; & 0 & 0 & 0\\ \; & \; & & \; & & 0 & 0\\ \mathrm{sym}. & \; & & \; & & \; & 0 \end{array}\right). \end{array}$$

For the derivation of $V_{\theta }^{\left(1\right)}$, let $zc=\left\{1,{\left(z-c\right)}^{+},z\right\}$ and $xt=\left\{1,{\left(x-t\right)}^{+},x\right\}$. Since the matrix $V_{\theta }^{\left(1\right)}$ is symmetric, we only need to derive the upper diagonal elements. The first row of $V_{\theta }^{\left(1\right)}$ is is the row concatenation of $\partial^{2}E_{XYZ}\ell \left(\theta \right)/\partial \alpha^{2}$, $\partial^{2}E_{XYZ}\ell \left(\theta \right)/\partial \alpha \partial \beta$, $\partial^{2}E_{XYZ}\ell \left(\theta \right)$/ ∂α∂c, $\partial^{2}E_{XYZ}\ell \left(\theta \right)/\partial \alpha \partial t$, $\partial^{2}E_{XYZ}\ell \left(\theta \right)/\partial \alpha \partial \rho$, $\partial^{2}E_{XYZ}\ell \left(\theta \right)/\partial \alpha \partial \sigma_{u}$, and $\partial^{2}E_{XYZ}\ell \left(\theta \right)/\partial \alpha \partial \sigma_{v}$, such that

$$V_{1,\theta }^{\left(1\right)}=\frac{1}{\left(1-\rho^{2}\right)}E_{XYZ}\left[{\left(zc\right)}^{T}\left\{-\frac{zc}{\sigma_{v}^{2}},\frac{\rho xt}{\sigma_{v}\sigma_{u}},\frac{\alpha I(z>c)}{\sigma_{v}^{2}},\frac{-\rho \beta I(x>t)}{\sigma_{v}\sigma_{u}},\frac{2\rho v}{\sigma_{v}^{2}\left(1-\rho^{2}\right)}-\frac{u(1+\rho^{2})}{\left(1-\rho^{2}\right)\sigma_{v}\sigma_{u}},\frac{\rho u}{\sigma_{v}\sigma_{u}^{2}},\frac{\rho u}{\sigma_{v}^{2}\sigma_{u}}-\frac{2v}{\sigma_{v}^{3}}\right\}\right].$$

The second row of $V_{\theta }^{\left(1\right)}$ is the row concatenation of $\partial^{2}E_{XYZ}\ell \left(\theta \right)/\partial \beta^{2}$, $\partial^{2}E_{XYZ}\ell \left(\theta \right)/\partial \beta \partial c$, $\partial^{2}E_{XYZ}\ell \left(\theta \right)/\partial \beta \partial t$, $\partial^{2}E_{XYZ}\ell \left(\theta \right)/\partial \beta \partial \rho$, $\partial^{2}E_{XYZ}\ell \left(\theta \right)/\partial \beta \partial \sigma_{u}$, and $\partial^{2}E_{XYZ}\ell \left(\theta \right)/\partial \beta \partial \sigma_{v}$ such that

$$V_{2,\theta }^{\left(1\right)}=\frac{1}{\left(1-\rho^{2}\right)}E_{XYZ}\left[{\left(xt\right)}^{T}\left\{-\frac{xt}{\sigma_{u}^{2}},-\frac{\rho \alpha I(z>c)}{\sigma_{u}\sigma_{v}},\frac{\beta I(x>t)}{\sigma_{v}^{2}},\frac{2\rho u}{\sigma_{u}^{2}\left(1-\rho^{2}\right)}-\frac{v(1+\rho^{2})}{\left(1-\rho^{2}\right)\sigma_{v}\sigma_{u}},\frac{\rho v}{\sigma_{v}^{3}}-\frac{2u}{\sigma_{u}^{3}},\frac{\rho v}{\sigma_{u}\sigma_{v}^{2}}\right\}\right].$$

The third row of $V_{\theta }^{\left(1\right)}$ is the row concatenation of $\partial^{2}E_{XYZ}\ell \left(\theta \right)/\partial c^{2}$, $\partial^{2}E_{XYZ}\ell \left(\theta \right)/\partial c\partial t$, $\partial^{2}E_{XYZ}\ell \left(\theta \right)/\partial c\partial \rho$, $\partial^{2}E_{XYZ}\ell \left(\theta \right)/\partial c\partial \sigma_{u}$, and $\partial^{2}E_{XYZ}\ell \left(\theta \right)/\partial c\partial \sigma_{v}$ such that

$$V_{3,\theta }^{\left(1\right)}=\frac{1}{\left(1-\rho^{2}\right)}E_{XYZ}\left[{\left\{\alpha I(z>c)\right\}}^{T}\left\{\frac{\alpha I(z>c)}{\sigma_{v}\sigma_{u}},\frac{\beta I(x>t)}{\sigma_{u}^{2}},\frac{v(\rho^{2}+1)}{\sigma_{u}\sigma_{v}\left(1-\rho^{2}\right)}-\frac{2\rho v}{\sigma_{v}^{2}\left(1-\rho^{2}\right)},-\frac{\rho u}{\sigma_{v}\sigma_{u}^{2}},\frac{2v}{\sigma_{v}^{3}}-\frac{\rho u}{\sigma_{u}\sigma_{v}^{2}}\right\}\right].$$

The fourth row of $V_{\theta }^{\left(1\right)}$ is the row concatenation of $\partial^{2}E_{XYZ}\ell \left(\theta \right)/\partial t^{2}$, $\partial^{2}E_{XYZ}\ell \left(\theta \right)/\partial t\partial \rho$, $\partial^{2}E_{XYZ}\ell \left(\theta \right)/\partial t\partial \sigma_{u}$, and $\partial^{2}E_{XYZ}\ell \left(\theta \right)/\partial t\partial \sigma_{v}$ such that

$$\begin{array}{c} V_{4,\theta }^{\left(1\right)}=\frac{1}{\left(1-\rho^{2}\right)}E_{XYZ}\left[{\left\{\beta I(x>t)\right\}}^{T}\left\{-\frac{\beta I(x>t)}{\sigma_{v}^{2}},\frac{v(1+\rho^{2})}{\sigma_{v}\sigma_{u}\left(1-\rho^{2}\right)}-\frac{2\rho u}{\left(1-\rho^{2}\right)\sigma_{u}^{2}},\frac{2u}{\sigma_{v}^{3}}-\frac{\rho v}{\sigma_{v}\sigma_{u}^{2}},-\frac{\rho v}{\sigma_{u}\sigma_{v}^{2}}\right\}\right]. \end{array}$$

The remaining terms in $V_{\theta }^{\left(1\right)}$ is given by

$$\partial^{2}E_{XYZ}\ell \left(\theta \right)/\partial \rho^{2}=\frac{1+\rho^{2}}{{\left(1-\rho^{2}\right)}^{2}}-\frac{4uv\rho (\rho^{2}+1)}{\sigma_{u}\sigma_{v}{\left(\rho^{2}-1\right)}^{3}}+\frac{2\rho uv}{\sigma_{u}\sigma_{v}{\left(\rho^{2}-1\right)}^{2}},$$

$$\partial^{2}E_{XYZ}\ell \left(\theta \right)/\partial \rho \partial \sigma_{u}=\frac{2\rho u^{2}}{{\left(1-\rho^{2}\right)}^{2}\sigma_{u}^{3}}-\frac{2uv\rho^{2}}{\sigma_{u}^{2}\sigma_{v}{\left(\rho^{2}-1\right)}^{2}}-\frac{uv}{\sigma_{u}^{2}\sigma_{v}\left(1-\rho^{2}\right)},$$

$$\partial^{2}E_{XYZ}\ell \left(\theta \right)/\partial \rho \partial \sigma_{v}=\frac{2\rho v^{2}}{{\left(1-\rho^{2}\right)}^{2}\sigma_{v}^{3}}-\frac{2uv\rho^{2}}{\sigma_{u}\sigma_{v}^{2}{\left(\rho^{2}-1\right)}^{2}}-\frac{uv}{\sigma_{u}\sigma_{v}^{2}\left(1-\rho^{2}\right)},$$

$$\partial^{2}E_{XYZ}\ell \left(\theta \right)/\partial \sigma_{u}^{2}=\frac{2\rho uv}{\left(1-\rho^{2}\right)\sigma_{v}\sigma_{u}^{3}}-\frac{3u^{2}}{\sigma_{u}^{4}\left(1-\rho^{2}\right)}+\frac{1}{\sigma_{u}^{2}},$$

$$\partial^{2}E_{XYZ}\ell \left(\theta \right)/\partial \sigma_{u}\sigma_{v}=\frac{\rho uv}{\left(1-\rho^{2}\right)\sigma_{v}^{2}\sigma_{u}^{2}},$$

$$\partial^{2}E_{XYZ}\ell \left(\theta \right)/\partial \sigma_{v}^{2}=\frac{2\rho uv}{\left(1-\rho^{2}\right)\sigma_{u}\sigma_{v}^{3}}-\frac{3v^{2}}{\sigma_{v}^{4}\left(1-\rho^{2}\right)}+\frac{1}{\sigma_{v}^{2}}.$$

In terms of the matrix $V_{\theta }^{\left(2\right)}$, we decompose the following elements

$$\begin{array}{c} V_{\alpha c}^{\left(2\right)}=E_{XYZ}\left[\frac{v}{\sigma_{v}^{2}\left(1-\rho^{2}\right)}-\frac{\rho u}{\sigma_{u}\sigma_{v}\left(1-\rho^{2}\right)}\times \left(\begin{array}{cccc} 0 & \cdots & \cdots & 0\\ -I(z>c_{1}) & 0 & \cdots & 0\\ \vdots & \; & & \vdots \\ 0 & \cdots & \cdots & -I(z>c_{K})\\ 0 & \cdots & \cdots & 0 \end{array}\right)\right], \end{array}$$

$$\begin{array}{c} V_{\beta t}^{\left(2\right)}=E_{XYZ}\left[\frac{u}{\sigma_{u}^{2}\left(1-\rho^{2}\right)}-\frac{\rho v}{\sigma_{u}\sigma_{v}\left(1-\rho^{2}\right)}\times \left(\begin{array}{cccc} 0 & \cdots & \cdots & 0\\ -I(x>t_{1}) & 0 & \cdots & 0\\ \vdots & \; & & \vdots \\ 0 & \cdots & \cdots & -I(x>t_{J})\\ 0 & \cdots & \cdots & 0 \end{array}\right)\right], \end{array}$$

$$\begin{array}{c} V_{cc}^{\left(2\right)}=E_{XYZ}\left[\frac{1}{\sigma_{u}\sigma_{v}\left(1-\rho^{2}\right)}\right]\times \left(\begin{array}{cccc} -\alpha_{1}f_{Z}\left(c_{1}\right) & 0 & \cdots & 0\\ \vdots & \; & & \vdots \\ 0 & \cdots & \cdots & -\alpha_{K}f_{Z}\left(c_{K}\right) \end{array}\right), \end{array}$$

$$\begin{array}{c} V_{tt}^{\left(2\right)}=E_{XYZ}\left[\frac{1}{\sigma_{v}^{2}\left(\rho^{2}-1\right)}\right]\times \left(\begin{array}{cccc} \beta_{1}f_{X}\left(t_{1}\right) & 0 & \cdots & 0\\ \vdots & \; & & \vdots \\ 0 & \cdots & \cdots & \beta_{J}f_{X}\left(t_{J}\right) \end{array}\right). \end{array}$$

It is easy to check that when the model is correctly specified, $V_{\theta }^{\left(2\right)}=0$ and $V_{\theta }=-E_{XYZ}\left\{\dot{\ell }\left(\theta \right)\dot{\ell }{\left(\theta \right)}^{T}\right\}$.

## Appendix B. Theorems

Define Pf as the expectation Ef(X)=∫fdP and abbreviate the average $n^{-1}\sum_{i=1}^{n}f\left(X_{i}\right)$ to $P_{n}f$, an empirical distribution. Furthermore, we define

$$M_{n}\left(\theta \right)=1/n\sum_{i=1}^{n}m_{\theta }\left(X_{i}\right)=P_{n}m_{\theta }\;\mathrm{and}\;\Psi_{n}\left(\theta \right)=1/n\sum_{i=1}^{n}\psi_{\theta }\left(X_{i}\right)=P_{n}\psi_{\theta }.$$

**Theorem** **A1** (Theorem 5.7 of van der Vaart [28]). *Let $M_{n}$ be random functions and let M be a fixed function of θ such that for every ϵ>0*

$$\begin{array}{cc} & \underset{\theta \in \Theta }{\sup}\left|M_{n}\left(\theta \right)-M\left(\theta \right)\right|\overset{P}{\to }0,\\ & \underset{\theta :d\left(\theta ,\theta_{0}\right)\geq \varepsilon }{\sup}M\left(\theta \right)<M\left(\theta_{0}\right). \end{array}$$

*Then every sequence of estimators ${\hat{\theta }}_{n}$ with $M_{n}\left({\hat{\theta }}_{n}\right)\geq M_{n}\left(\theta_{0}\right)-o_{P}\left(1\right)$ converges in probability to $\theta_{0}$.*

**Theorem** **A2** (Theorem 5.14 of van der Vaart [28]). *For each θ in an open subset of Euclidean space, let $\theta \mapsto \psi_{\theta }\left(x\right)$ be twice continuously differentiable for every x. Suppose that $P\psi_{\theta_{0}}=0$, that $P∥\psi_{\theta_{0}}{∥}^{2}<\infty$ and that the matrix $P{\dot{\psi }}_{\theta_{0}}$ exists and is nonsingular. Assume that the second-order partial derivatives are dominated by a fixed integrable function $\ddot{\psi }\left(x\right)$ for every θ in a neighborhood of $\theta_{0}$. Then every consistent estimator sequence ${\hat{\theta }}_{n}$ such that $\Psi_{n}\left({\hat{\theta }}_{n}\right)=0$ for every n satisfies*

$$\sqrt{n}\left({\hat{\theta }}_{n}-\theta_{0}\right)=-{\left(P{\dot{\psi }}_{\theta_{0}}\right)}^{-1}\frac{1}{\sqrt{n}}\sum_{i=1}^{n}\psi_{\theta_{0}}\left(X_{i}\right)+o_{P}\left(1\right).$$

*In particular, the sequence $\sqrt{n}\left({\hat{\theta }}_{n}-\theta_{0}\right)$ is asymptotically normal with mean zero and covariance matrix ${\left(P{\dot{\psi }}_{\theta_{0}}\right)}^{-1}P\psi_{\theta_{0}}\psi_{\theta_{0}}^{T}{\left(P{\dot{\psi }}_{\theta_{0}}\right)}^{-1}$.*

## Appendix C. Additional Simulation Results

**Table A1:** Empirical biases, theoretical standard errors (tse), and empirical standard errors (ese) of $\hat{\theta }$, as well as 95% coverage probabilities (cp) on θ for scenario 1 with sample size 1000.

	ρ=0.2				ρ=0.5				ρ=0.8			
	bias	tse	ese	cp	bias	tse	ese	cp	bias	tse	ese	cp
$\alpha_{0}$	−8.30	27.04	29.78	928	−6.48	25.41	27.35	933	−3.28	22.00	22.78	942
$\alpha_{1}$	3.08	68.29	70.76	950	2.96	64.99	67.54	932	2.46	53.88	55.17	949
$\alpha_{2}$	−7.90	30.92	32.50	936	−6.05	28.90	30.11	938	−2.79	23.05	23.46	955
$\beta_{0}$	−3.61	38.76	39.70	949	−2.80	36.66	37.62	938	−1.77	30.74	31.19	945
$\beta_{1}$	−0.46	55.44	54.45	956	−0.18	52.00	51.80	948	0.65	41.79	42.11	939
$\beta_{2}$	−1.21	24.18	24.78	938	−0.88	22.76	23.35	928	−0.43	18.38	18.42	949
*c*	−41.08	123.92	167.07	873	−31.07	111.23	148.14	873	−12.70	79.47	98.09	886
*t*	−7.63	68.18	76.36	919	−4.90	61.13	66.47	920	−1.50	43.69	46.31	935
ρ	1.08	34.23	34.63	948	1.08	26.53	26.77	948	0.72	12.41	12.49	946
$\sigma^{2}$	−0.86	9.82	9.68	949	−0.64	10.96	10.75	949	−0.26	12.68	12.42	946

Note: all numbers are multiplied by 1000. These results are based on 1000 replications.

**Table A2:** Empirical biases, theoretical standard errors (tse), and empirical standard errors (ese) of $\hat{\theta }$, as well as 95% coverage probabilities (cp) on θ for scenario 2 with sample size 1000.

	ρ=0.2				ρ=0.5				ρ=0.8			
	bias	tse	ese	cp	bias	tse	ese	cp	bias	tse	ese	cp
$\alpha_{0}$	−25.84	176.86	168.03	943	−15.74	155.65	161.09	929	−7.23	104.42	114.68	927
$\alpha_{1}$	8.53	115.79	106.55	956	7.00	103.98	101.28	947	4.01	73.82	75.63	944
$\alpha_{2}$	8.49	112.53	105.08	964	5.55	108.98	107.91	958	3.31	98.38	93.69	957
$\alpha_{3}$	−11.29	108.14	99.84	951	−5.51	95.98	95.87	934	−1.84	67.37	71.38	935
$\beta_{0}$	−2.87	83.31	84.73	942	−2.03	77.04	78.41	945	−1.09	59.84	62.78	929
$\beta_{1}$	3.86	49.69	50.23	945	2.72	46.32	46.96	942	2.34	36.27	37.33	941
$\beta_{2}$	5.77	72.64	67.82	960	3.88	67.56	63.16	963	2.32	53.65	52.82	939
$\beta_{3}$	−0.69	39.92	40.14	940	-0.48	37.14	37.55	943	−0.11	29.14	31.00	944
$c_{1}$	−16.09	171.89	185.99	923	0.96	152.10	212.49	907	2.67	103.26	158.39	891
$c_{2}$	−2.69	81.76	95.51	912	4.37	75.10	125.84	903	7.86	56.60	131.88	894
$t_{1}$	2.18	53.28	57.74	933	2.08	47.82	52.28	921	2.33	34.17	38.55	921
$t_{2}$	20.13	111.57	136.46	925	13.61	99.30	108.45	930	13.30	70.22	85.85	927
ρ	1.21	32.96	33.18	953	1.52	25.63	25.73	950	0.93	12.14	12.31	942
$\sigma^{2}$	−1.41	9.81	9.64	948	−1.17	10.88	10.59	951	−0.58	12.57	12.27	948

Note: all numbers are multiplied by 1000. These results are based on 1000 replications.
